# Implementing an online pharmaceutical service using design science research

**DOI:** 10.1186/s12911-017-0428-2

**Published:** 2017-03-27

**Authors:** Luís Velez Lapão, Miguel Mira da Silva, João Gregório

**Affiliations:** 1 Global Health and Tropical Medicine (GHTM), WHO Collaborating Center for Health Workforce Policy and Planning, Instituto de Higiene e Medicina Tropical, Universidade Nova de Lisboa, Rua da Junqueira, n° 100, Lisboa, 1349-008 Portugal; 2 Instituto Superior Técnico, Universidade de Lisboa, Avenida Rovisco Pais, 1, Lisboa, 1049-001 Portugal

**Keywords:** Pharmaceutical services, Services implementation, Online services, Patient experience, Design science research

## Abstract

**Background:**

The rising prevalence of chronic diseases is pressing health systems to introduce reforms. Primary healthcare and multidisciplinary models have been suggested as approaches to deal with this challenge, with new roles for nurses and pharmacists being advocated. More recently, implementing healthcare based on information systems and technologies (e.g. eHealth) has been proposed as a way to improve health services. However, implementing online pharmaceutical services, including their adoption by pharmacists and patients, is still an open research question. In this paper we present ePharmacare, a new online pharmaceutical service implemented using Design Science Research.

**Methods:**

The Design Science Research Methodology (DSRM) was chosen to implement this online service for chronic diseases management. In the paper, DSRM’s different activities are explained, from the definition of the problem to the evaluation of the artifact. During the design and development activities, surveys, observations, focus groups, and eye-tracking glasses were used to validate pharmacists’ and patients’ requirements. During the demonstration and evaluation activities the new service was used with real-world pharmacists and patients.

**Results:**

The results show the contribution of DSRM in the implementation of online services for pharmacies. We found that pharmacists spend only 50% of their time interacting with patients, uncovering a clear opportunity to implement online pharmaceutical care services. On the other hand, patients that regularly visit the same pharmacy recognize the value in patient follow-up demanding to use channels such as the Internet for their pharmacy interactions. Limitations were identified regarding the high workload of pharmacists, but particularly their lack of know-how and experience in dealing with information systems (IST) for the provision of pharmaceutical services.

**Conclusions:**

This paper summarizes a research project in which an online pharmaceutical service was proposed, designed, developed, demonstrated and evaluated using DSRM. The main barriers for pharmacists’ adoption of online pharmaceutical services provision were the lack of time, time management and information systems usage skills, as well as a precise role definition within pharmacies. These problems can be addressed with proper training and services reorganization, two proposals to be investigated in future works.

## Background

Chronic diseases are already the main cause of mortality in Europe, and are becoming a challenge for middle and low income economies due to the demographic and epidemiological transitions taking place [1]. The increasing prevalence of chronic diseases is inducing health system reforms with interprofessional collaboration in primary care emerging as a model of integrated healthcare service provision [2, 3]. The goal of these multidisciplinary models is to transform the daily care of chronic patients, assigning major roles for non-physicians, such as community pharmacists (CP) and nurses [4–6].

The focus on multidisciplinary models of care and the consequent primary healthcare reforms are encouraging the rethinking of community pharmacists’ role [7]. Community Pharmacy orientation towards a patient-centered practice has become the new paradigm of pharmacy practice, supported by the development of a clinical role for community pharmacists and a more active role for patients in their own disease management [8, 9]. This new paradigm has led to the development of new “pharmaceutical services” concepts [10].

There are a number of different services performed by pharmacists in different countries that match CP’s role in primary healthcare. These services range from the traditional distribution services to more advanced disease management services [11, 12]. Moreover, pharmaceutical services have been considered extremely valuable for professionals, patients and healthcare systems, mainly due to a greater efficiency and improvement in individual patient’s health related outcomes [4, 9, 13].

Costa and colleagues [14] have described the status and outlined the trends of community pharmacy services in Portugal. CP in Portugal work exclusively in independent pharmacies, since large chains are not allowed. The opening of community pharmacies is regulated by the national medicines regulatory agency (INFARMED). Pharmacies have a National Health Service (NHS) contract for dispensing prescription medicines, with legislation establishing medicines’ profit margins and patients’ co-payments [15] Over the last twenty years Portuguese community pharmacies have developed consultation services to manage chronic patients and their therapies, in line with international developments on “pharmaceutical services” [14, 16].

Good communication between professionals is essential in multidisciplinary practice, making Information Systems and Technologies (IST) a prerequisite underpinning healthcare services in future health systems [17]. The continuous development of IST led to the onset of eHealth, that can be defined as “the utilization of IST to support health service provision, complying with the needs of citizens, patients, health professionals and other providers” [18]. IST in healthcare have been used primarily to improve administrative management. However, implementation of eHealth services has the potential to promote a better access to information by patients and providers, improve the quality, efficacy and safety of healthcare services, and encourage healthier lifestyles [19–21].

Investing in IST and modernizing the architecture of pharmacies is considered as a necessary and critical step towards the diffusion of new forms of practice [22, 23]. eHealth services may develop in the next few years to harvest the full potential of CP, enhancing their role in the primary care network and supporting their activities in chronic disease management [24].

However, difficulties in eHealth implementation are frequently reported [25, 26]. Most of these difficulties are frequently attributed to managerial and behavioral factors [27]. To resolve these difficulties, some authors propose that a user centered approach should be promoted to be certain that eHealth services will satisfy user’s needs [28]. The user centered approach also reinforce users’ ownership of the system leading to higher compliance and ongoing use of the system [29].

The complex characteristics of the problems affecting health systems worldwide demand new ideas and innovations, aiming at more responsive healthcare services [30]. The implementation of multidisciplinary services models requires proper planning and management, especially when innovation experience and patient knowledge is lacking. The ePharmacare project was designed considering all these challenges. The main goal of this project was to explore the development of eHealth used to address the challenges of community pharmacists’ integration with primary healthcare services. Additionally, the project would also assess the potential of eHealth in the provision of pharmaceutical services by actively enabling the interaction with patients and promoting their education. Furthermore, the project is expected to address the use of eHealth supporting health services, establishing its acceptability, feasibility, sustainability, and adaptability to future changes.

In order to achieve these objectives, an online pharmaceutical service was conceived, designed, developed, demonstrated and evaluated using the Design Science Research Methodology (DSRM) [31, 32]. This service (e.g. an artifact for DSRM) would support patients’ disease and therapeutic monitoring by a CP. Patients were enrolled in an active way, advising them to use the service to interact with pharmacists. Information about patients can be used to understand patients’ treatment effects and success rate, but that information is also valuable for assessing the service acceptability and usability.

After this introduction, the paper is divided in three sections. In the next section, we highlight the research method used for designing, demonstrating and evaluating the service. Results section presents the results according to the activities that support DSRM [31]. Discussion section analyzes and discusses the results in more detail. The paper then closes with a conclusion that includes the main lessons learned plus future work.

## Methods

The implementation of online services in a healthcare practice presents several challenges [25]. The design of the service must be adequate to both practitioners and users, as well as cost-effective. On the other hand, the utilization of an online health service has the potential to impact user behavior and perceived usefulness [20]. Therefore, there is a need to use a research method that takes into account the interaction between health professionals and the end user.

The Design Science Research Methodology (DSRM) [31, 32] was chosen since DSRM has demonstrated its ability to study the connection between research and professional practices by designing, implementing and evaluating artifacts that address a specific need. In fact, DSRM studies the artificial - any phenomena created by humans - in a rigorous process of proposing artifacts to solve problems, evaluating what has been projected or what is functioning, and communicating the obtained results [33].

The main output of a DSRM project must be an artifact. In this case, the artifact will be an eHealth pharmaceutical service. Hevner and colleagues [32] have established guidelines for a consequent DSRM study. Later, Peffers and colleagues [31] proposed converting those guidelines into six activities. For the research presented in this paper, these six activities had a specific set of tasks (Table 1).

**Table 1:** DSRM activities and respective tasks

DSRM activity	Tasks
1. Identify problem & motivate	Setup the context using a scenario design for Portuguese pharmacists in 2020. Online survey of IST utilization in Portuguese pharmacies. Observational time-and-motion study to assess pharmacists’ current work patterns and potential demand for pharmaceutical care services Cost of current pharmaceutical services
2. Define objectives of a solution	Set of qualitative interviews performed within primary health centers and hospitals
3. Design & development	Design of artifact (online service based on a software platform) for pharmaceutical care provision
4. Demonstration	Field study to test the platform in two settings with a selected group of patients
5. Evaluation	The usability of the platform was assessed through the use of “task scenarios” with eye-tracking glasses and semi-structured interviews of the participants
6. Communication	Practitioners, conference communications, journal papers, and theses.

In the remaining of this section, an explanation of the tasks performed in each activity is provided in more detail.

### Activity 1 - Identify problem & motivate

In order to setup the context for this research, a scenario planning exercise was performed to design future scenarios for Portuguese community pharmacists. The exercise recognized the changing environment as a huge challenge but also an opportunity to develop the role that community pharmacists may play in the Portuguese health system. For this exercise two objectives were considered: (i) to analyze the possible evolution of community pharmacists’ role in the Portuguese healthcare system by building and studying three different scenarios, and (ii) to identify the main driving forces and related uncertainties that may impact on the definition of the future community pharmacists’ role [23].

Furthermore, an online survey was launched to obtain a general overview of the current state of pharmaceutical services provision and the IST usage in community pharmacies, based on a previously developed survey performed in Switzerland [34]. The survey was translated and validated with two academic researchers specialized on Portuguese community pharmacies. The survey was implemented using Google® Forms, and then sent via email to 323 pharmacies members of the Association of Portuguese Pharmacies (AFP).

Following this online survey, an observational time-and-motion study was performed to study pharmacists’ work patterns and pharmaceutical care services’ potential demand [35, 36]. The observational study was performed in four pharmacies of the metropolitan Lisbon area, during an eight hours full shift. The objective was to understand the potential value of an online pharmaceutical service, and how that service might fit in a pharmacist’s workday [37]. The study characterized the activities performed by CP, including the entire array of provided services, time spent with patients, and an estimation of chronic patients’ yearly visits to a pharmacy. Using the main outputs of the observational study, such as the time spent performing a service and the activities required to carry out each service, it was possible to determine the costs of current pharmaceutical services being provided, through a Time-driven Activity Based Cost analysis [38]. After finding all the activity cost-rates, it is possible to determine the cost of a specific service, using the following time equation (where *t*_*n*_ is the average time spent in each activity necessary to a certain service):

$$ \mathrm{Service}\ \mathrm{cost} = \left({\mathrm{t}}_1*\mathrm{cost}\ {\mathrm{rate}}_1\right) + \left({\mathrm{t}}_2*\mathrm{cost}\ {\mathrm{rate}}_2\right) + \left({\mathrm{t}}_3*\mathrm{cost}\ {\mathrm{rate}}_3\right) + \left({\mathrm{t}}_{\mathrm{n}}*\mathrm{cost}\ {\mathrm{rate}}_{\mathrm{n}}\right) $$

### Activity 2 - Defining objectives of a solution

The aim of this activity was to identify the services required by patients when they interact with pharmacies. The identification of those services, helped to define features of the online service. The final objective of this activity was to inform the design of an online service with functionalities that potential customers felt lacking in their community pharmacy experience, added to functionalities that could mimic the work processes of the Dáder methodology for pharmaceutical care provision [39]. A set of 50 semi-structured interviews focusing on customer service were performed, with patients at health centers and hospitals in Lisbon and Oporto [40]. The interview guide was developed with the aim of gathering opinions regarding the current service concept offered by community pharmacies, as well as what customers would like to have within existing or new services [41]. These interviews also identified the most critical service failures that customers experienced, and collected feedback about potential solutions. The interviewees were selected among patients of different age groups in the waiting rooms of these health services, after their consent to participate was obtained verbally by the independent researcher following a brief explanation of the interview’s objective. The interviews were performed in person and audio-recorded, following an interview guide previously developed.

### Activity 3 - Design & development

The design and development of the online pharmaceutical service was based on the Dáder methodology for pharmaceutical care and Service Experiment Blueprint concepts, enabling the design of service characteristics and functionalities required by chronic patients [39, 42]. Service Experience Blueprint (SEB) ensures a customer driven design able to co-create value with end customers [42, 43]. The SEB method enhances the design of customer experiences, especially for technology-enabled services and contributes for a stronger focus on customer-firm relationship. Modelling methodologies can also provide an additional understanding of the dynamics of an organization to better align pharmacy services’ design with operations [44].

The design and development of the software platform (that supports the online service) was based on the Agile methodology, [45, 46] using “sprints” of seven days managed on Trello® online service. After the end of each sprint, a new version of the software platform was published in order to be immediately used by pharmacists and patients. This methodology permits the collection of feedback from end users almost continuously, so that new features are prioritized in order to extract maximum value from the service.

### Activity 4 - Demonstration

In this activity, the utility (for patients and pharmacists) of the proposed online service was demonstrated. The online service was tested in two different settings during nine months: (i) three CP working within three of the observational study’s community pharmacies using the service and face-to-face consultations to interact with patients (Pharmacists “P”); (ii) a CP using the service and scheduled meetings interacted with patients without the possibility of dispensing medicines (Pharmacist “C”). The main criteria to select pharmacists among the pharmacy’s staff was their ability and certification to provide pharmaceutical care services. Pharmacist “C” used the platform outside the community pharmacy to recruit patients among people of a Senior Citizens University in a Lisbon municipality and was also a certified pharmacist. A Senior Citizen University is a service provided by some municipalities, sometimes supported by official schools, where seniors (people with more than 65 years of age) go to pass their time and have learning activities. Among those activities, there usually are some Information technology classes.

The target users were purposively selected among patients that presented at least one prescription for chronic medicines and were active users of the internet. Patients were recruited during the first two months of the study according to the following criteria: to have a prescription of a hypertensive, antidiabetic or anti-cholesterol medicine; to have more than 65 years of age; to be an active Internet user. A survey was applied to determine patient’s internet utilization patterns. Pharmacist’s intervention included: patient education about medicines, completion of a drug therapy profile and drug history, assessment of initial drug compliance and patient counseling about lifestyle modifications. Initial training was given both to pharmacists and patients, and follow-up training was provided when needed.

### Activity 5 - Evaluation

The evaluation took place eight months after the start of the demonstration activity, and included tests with four pharmacists and three patients. The tests were based on a set of tasks executed by pharmacists and patients (Table 2). Eye-tracking glasses were used to determine which platform features were looked upon when performing each set of tasks and how long it took, in order to identify major bottlenecks in users’ experience of the platform. All testers were interviewed to provide more information about the utility of the online service and to identify missing features.

**Table 2:** Tasks developed for testing the online service

Pharmacist tasks	Patient tasks
Scenario I -. Enter the ePharmacare platform with your username and password. Look for the date of the next visit to your user XXX. Scenario II - Add a new medicine for the user XXX: Ben-u-ron 500 mg, 20 tablets; take one tablet after breakfast and one after dinner. Set the end date of package. Scenario III - Send a message to the user XXX: TEST. Scenario IV - Add the value of postprandial glucose of 182 mg/dL for the user XXX. Scenario V - Find the last blood pressure to the user XXX. Scenario VI - Arrange a visit for the user YYY for the day 07/20/2014, 10:00 am. Scenario VII - Verify the value of total cholesterol to user XXX. Scenario VIII - Verify that the user forum has calculated BMI.	Scenario I -. Enter the ePharmacare platform with your username and password. Seek new messages. Scenario II - Add the value of fasting glucose of 182 mg/dL. Scenario III – Add the blood pressure value 135/85 mm/Hg. Scenario IV – Add the height and weight values. Scenario V - Find the last blood pressure value. Scenario VI – Add a new medicine to the user profile: Ben-u-ron 1000 mg, 18 tablets'; take one tablet after breakfast and one after dinner. Scenario VII – Verify your next appointment with the Pharmacist. Scenario VIII – Send a message “TEST” to the CP. Scenario IX – Find the end date for the package of Ben-u-ron. Scenario X – You have been taking two cups of green tea daily, add this information to your profile.

### Activity 6 – Communication

The Communication activity aim at disseminating the project’s results. This activity was performed throughout the duration of the research project and consisted in oral communications, papers published in conference proceedings within the fields of study and three papers already published in peer reviewed journals [23, 37, 38].

### Data analysis

Statistical and other data analyses were performed with MS Excel® and SPSS® (Software Package for Social Sciences; Version 20, IBM, Chicago, IL). The content analysis of the qualitative interviews was performed with Computer Assisted Qualitative Data Analysis Software (CAQDAS) – QSR NVIVO® 10. All data were kept anonymous and confidential.

## Results

The utilization of DSRM’s six activities produced results which are presented in the following sub-sections. After these sections, a discussion section ensues with an overall discussion about the results, the consequences for community pharmacists and a critical appraisal of the DSRM.

### Activity 1: Identify problem and motivate

The problem identification activity is crucial to help focus the DSRM and to guarantee user involvement. Three complementary approaches were used to define the problem: a scenario exercise, an online survey, and an observational study.

- Scenario exercise

The scenario analysis highlighted that the development of a new role for community pharmacists is dependent on the economic and legislative environment in which these professionals operate and also of their inner ability to innovate and develop new services. In a scenario where online pharmaceutical services would be a reality across the country, new health regulations have to be in place alongside a more competitive pharmacy market. Besides that, an innovative approach by pharmacy managers and practicing CP will be needed.

b) Online survey

**Fig. 1:**
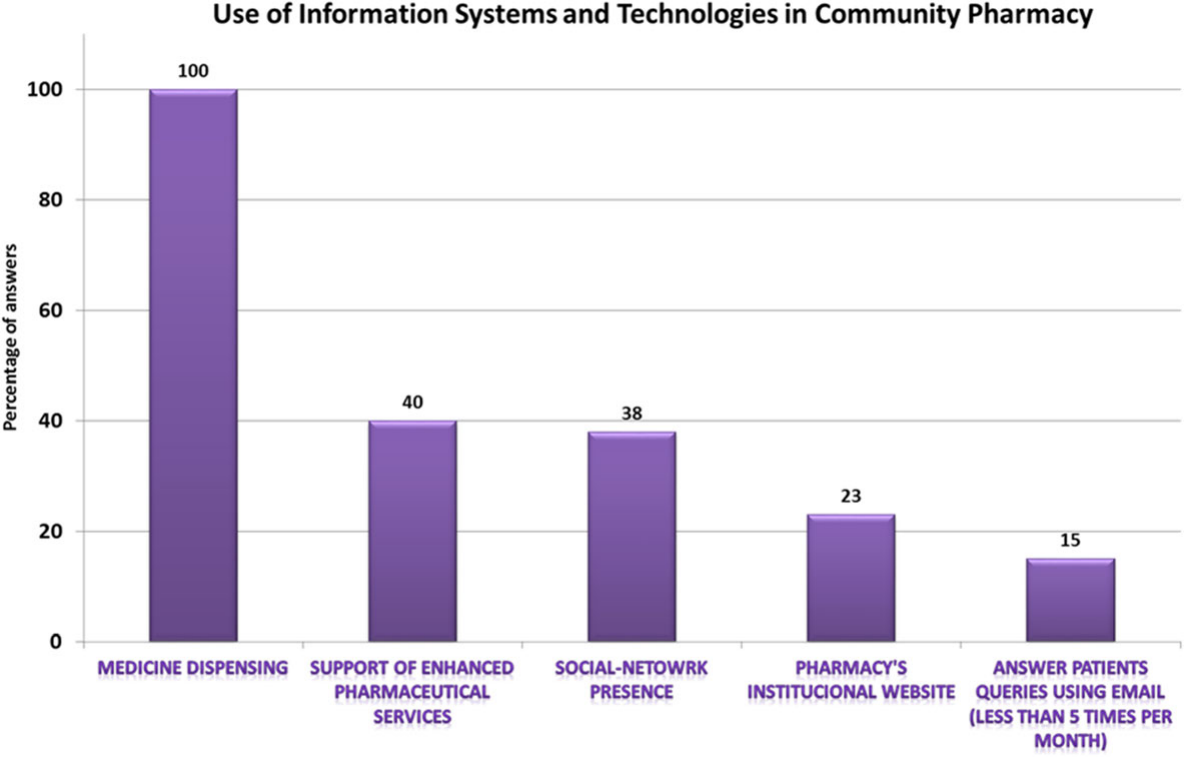
Characterization of Information Systems and Technologies use in a convenience sample ofPortuguese Community Pharmacies

The online survey had a low response rate (n = 16/323; 4.95%). In the respondent pharmacies, the average number of computers was 5 (min: 2; max: 10), with a ratio of 2.5 computer per pharmacy. All respondent pharmacies use IST for medicine dispensing and administrative tasks (stock management, reimbursement activities) (Fig. 1).

Approximately a quarter of respondent pharmacies have an Internet site and 38% have a social network presence. All respondent pharmacies claimed to check their email daily, although only 15% claim to have used the email to answer patients’ queries, and do so less than 5 times a month. To the question “what is the most important barrier to eHealth service provision in Pharmacy” all (100%) the respondents were unanimous in two options: the lack of financial incentives for online pharmaceutical services implementation and lack of support and guidance from professional organizations.

c) Observational study

**Fig. 2:**
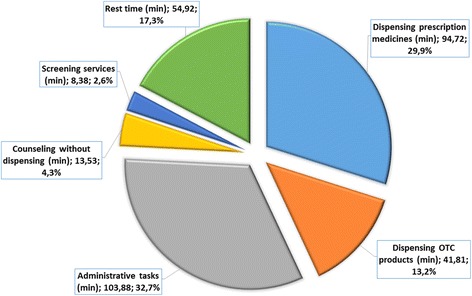
Average Percentage of daily time usage by pharmacists, based on a time-motion study in 4 pharmacies

The observational study took place during a week-day’s full 8 hours shift. In total, 16 pharmacists were observed. Eighty-five percent of the observed tasks were performed by pharmacists, equivalent to 65% of the total recorded time. Among the recorded tasks, between 77 and 85% had some sort of medicine dispensed. During the observation, professionals interacted on average with 40 customers, with 54% of the interactions occurring in the afternoon period (3 p.m. – 7 p.m.). About 54 minutes of free or idle time per pharmacist were found. However, the majority of the free time is spent in micro pauses, with 50% of the recorded breaks having less than 50 seconds while 11% were between 5 and 30 minutes in duration. Regarding the provision of pharmaceutical services, 29.9% of pharmacist time was spent dispensing prescriptions and 13.2% dispensing Over-the-counter (OTC) medicines. Also of relevance is the 4.3% of pharmacist time that was spent counselling patients without dispensing any medicine (Fig. 2).

Concerning other activities performed by pharmacists, 32.7% of pharmacists’ time was spent in administrative tasks, including ordering and storage of medicines, preparing prescriptions for reimbursement issues, and meetings with vendors and salespersons. In the recorded observations, the only service provided using an IST was the dispensing service. The IST was used to support sales, detecting possible interactions and identifying adverse effects of the dispensed medicines.

d) Costing study

In general, pharmaceutical services costs in the participant pharmacies were similar. The average cost for the dispensing service was €3.66; OTC dispensing average cost was €2.16; for the counselling service, the average cost was €1.34; Health screening services’ average costs were €3.59. The costing study also identified the most costly activities in pharmaceutical service provision. In these pharmacies, validation and dispensing of the prescription and the managing of inventory and other records, were found to be the highest-cost activities.

### Activity 2: Define objectives of a solution

After the problem identification activity, DSRM proposes the definition of objectives for a solution. To identify service requirements, a set of 50 qualitative interviews were performed. The qualitative research was undertaken in the hospitals of S. João (Porto) and Egas Moniz (Lisbon), and in the healthcare centers of Campanhã (Porto) and Ajuda (Lisbon). The interviewees were 24 males and 26 females (mean age of 44.4 years) that answered questions about community pharmacy services. 62% of the interviewees reported visiting a pharmacy at least once a month. 46% of the interviewees admitted that they sought healthcare provision with CP for minor issues before going to a general practitioner (GP). When asked about new pharmacy services, the home delivery and internet ordering of medicines were the most referred services, followed by integrating pharmacy services with primary care (e.g. gatekeeping, scheduling of GP consultations, patient follow-up).

Supported by the results from the first two activities, the main targets for the new service were now defined. The online pharmaceutical service should allow the pharmaceutical care of chronic patients without overloading community pharmacists’ workload, at a cost comparable to a screening service. This service should address the monitoring of patients’ therapy (covering medication reviews, medicine interactions, adherence and medication management), alongside features that aimed to test service integration with the primary healthcare (e.g. emission of reports to the GP).

### Activity 3: Design and development

From the insights obtained in the previous activities, the design of a disease management platform to support online pharmaceutical care services for chronic patients was proposed. The platform was called ePharmacare. The core of the platform is the storage of patient’s treatment information and the possibility to improve that information by allowing both pharmacists and patients to enter valuable information on the platform. Once pharmacists have their patients registered in the platform, it is possible to provide disease management on several levels: estimation of “re-fill dates”, providing the pharmacist with a prevision of when a re-stock and refill of medicines is needed for a specific patient; monitoring of therapeutic results; early detection of adverse reactions, addressing minor ailments or other queries reported by patients.

The built platform is able to calculate treatment end dates and warn the pharmacist when that date is approaching, while the patient will receive a notification email. The platform also offers patients the option to request their medicines online. This functionality supports pharmacists and patients on the control of patient’s therapeutic compliance and allows the streamlined just-in-time provision of medicines, either in-pharmacy or home delivered.

**Fig. 3:**
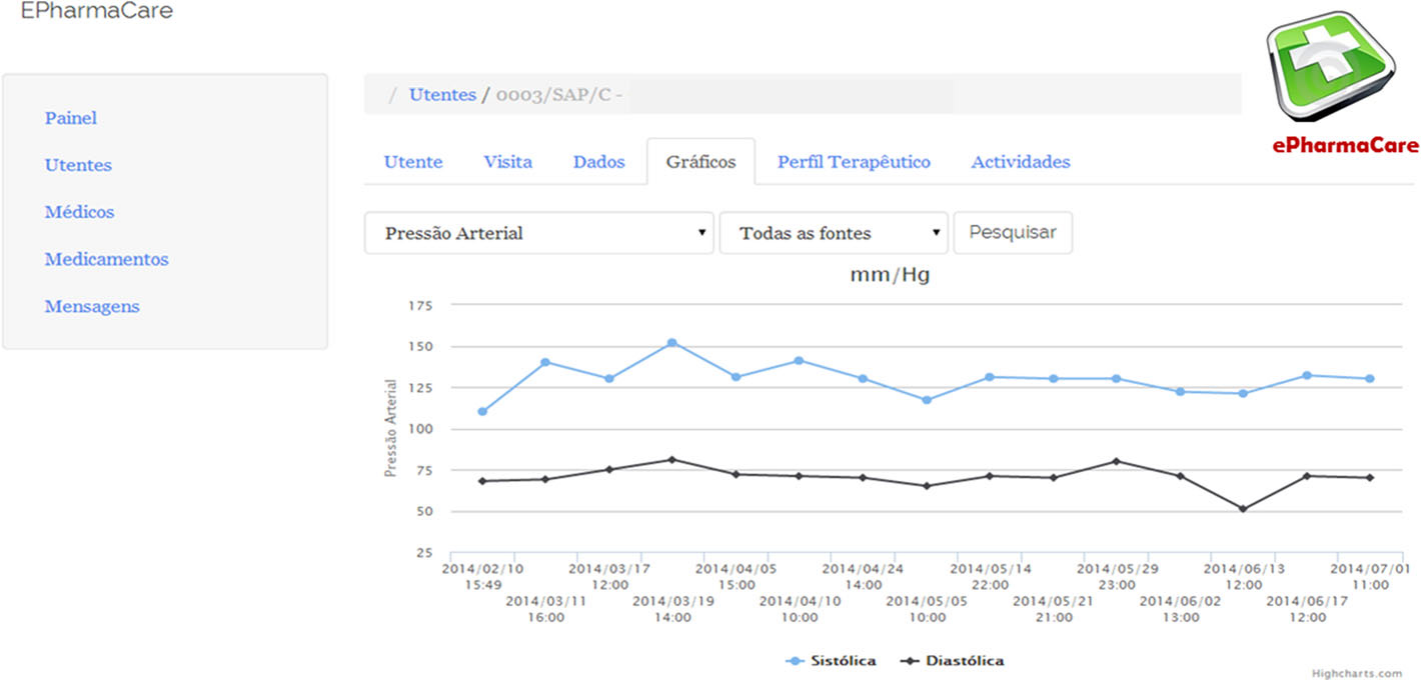
Screenshot of the blood pressure monitor (pharmacist view)

Allowing real-time monitoring of patient’s parameters such as blood pressure or glycaemia levels is another important aspect of the platform (Fig. 3). Currently, pharmacists monitor and register their patient’s therapeutic progress on paper (or not at all). ePharmacare allows patients and pharmacists to enter and store these data in a database. Pharmacists can then see, edit, organize, and interpret the data in a more convenient way. For patients, the potential of this feature lies in the possibility of accessing their own therapeutic and disease management data outside of the pharmacy, thus greatly improving their own decision making.

### Activity 4: Demonstration

A total of 28 patients showed interest in participating, answering the initial recruitment survey. Eighty-eight percent of these patients used the Internet for more than 2 years, mostly using the Internet to search for information regarding trips and holidays (73.1%). Only 57.7% of these patients claimed using the Internet to search for health related information.

Two Pharmacists “P” recruited three chronic patients each while another Pharmacist P recruited six patients. However, none of these patients used the platform more than once, being removed from the final analysis. Asked about the main reasons related with recruitment’s difficulty, Pharmacists “P” argued that they were too busy attending patients in the pharmacy. In their opinion, the process of recruiting patients was too long, taking about 30 minutes, including: brief explanation of the project (~10 minutes); patient answering the survey (~15 minutes); account creation (~5 minutes); and account verification and validation (~5 minutes). Also, they anticipated non-compliance with this type of intervention by their usual chronic patients, leading them to under-recruit.

Pharmacist “C” recruited 16 patients, but only 10 participated in the study during the full length of the demonstration. Pharmacist “C” patients held monthly meetings, observing, discussing and registering clinical data. These meetings finished with an information session about different health themes, but usually in the context of chronic diseases, such as the proper use of medications, interactions of medicines and herbal products, etc. The online information exchange between patients and Pharmacist “C” was related to questions of therapy modifications, self-administration of medicines or self-medication with OTC medicines and herbal products.

Among the collected parameters, the blood pressure and post-prandial glycaemia showed significant improvements (see Table 3). However, due to the low number of participants and the exploratory nature of the demonstration study, such improvements must be interpreted with caution.

**Table 3:** Physiological and biochemical parameters that showed improvement

Parameter	Beginning (Median [p25-p75])	End (Median [p25-p75])	P-value*
Systolic BP (mmHg)	167 [151 – 172]	157 [137 – 164]	0.008
Diastolic BP (mmHg)	74 [71 – 80]	70 [60 – 80]	0.028
Postprandial glycaemia (mg/dl)	257 [200 – 296]	199 [176 – 220]	0.028

*Wilcoxon signed-rank test; *p-*value < 0.05

### Activity 5: Evaluation

The objectives of this activity were to assess the usability of the platform and to identify missing features in the service, aiming to inform the next cycle of DSRM. To help in assessing the usability of the web platform, eye-tracking glasses were used. The tasks were performed while the glasses recorded a video which was analyzed afterwards.

It was found that a CP took an average of 7:38 minutes to perform the tasks. This is an important finding since it fits within the free time found in activity one. Nevertheless, it is more important to highlight that to really use this free time in an efficient way, some reorganization of pharmacies’ internal working processes and role-definition among professionals must be done. For patients, the time to perform a set of tasks was considered less relevant since patients can use the platform whenever is most convenient. The difficulties in using IST are common among this age group and are usually addressed in usability field studies [47]. Furthermore, the tasks were performed under the assistance of one researcher that offered help by pointing at the right tabs and menus when necessary. Given that this help should not be needed, the necessity to improve the design of the platform and the training given to patients were the first major outcomes of this evaluation.

After the eye-tracking evaluation, a semi-structured interview followed. In this interviews, we found that to maintain patient’s motivation to use the platform, periodic meetings or consultations have to be performed. Only when these meetings were regularly scheduled, patients used the platform to register their parameters or communicate with the pharmacist. Patients also found that one of the most valuable assets is the platform’s capability to assemble patient’s therapeutic profile and to calculate when each medicine will be finished or refilled, based on the dose, package size and treatment duration.

The main evaluation conclusions were the following:

- Pharmacists used the ePharmacare platform without difficulty during the usability test; Patients, however, needed some guidance, hinting at some design flaws and insufficient training.
- The addition of a dashboard is needed on the front page to simplify the access to information. This was felt by both pharmacists and patients. The font size was found to be too small for most patients, and not suitable for the standard screen resolution of pharmacies’ current information system;
- Patient’s main request was to have a “text box” to add information about non-conventional therapy, and the possibility to edit information. Also mentioned was the need to have a message system that allowed file attachment (e.g. clinical analysis results)
- Patients also asked for “messages” to alert them for therapy ending as well as to screening values out of the ordinary or above the established objectives;
- Some pharmacists found the “send a message” and “schedule a visit” features (which also allows sending a message) confusing;
- In pharmacist’s opinion, the main barriers to patient recruitment were: advanced age of patients (65-80 years); lack of time; lack of an adequate space to practice pharmaceutical care supported by the platform; populations’ lack of awareness; and patients refusal to share their data with a pharmacy;
- Patient’s demonstrated satisfaction with the platform and would like to continue to use it in their daily routine. They found the platform comfortable and simple to use, despite the design issues
- The lack of communication with a GP was felt by most of the patients and by all of the pharmacists. The communication with a GP feature was not implemented in this study since the goal was to address patients’ usability issues first. However, these suggestions will be implemented in the next research cycle.

## Discussion

The development of a new online pharmaceutical service could be an important contribution to efficient chronic diseases’ management. In this paper, a method to develop and implement a platform for online pharmaceutical services provision was proposed. Although the research work reported in this paper is exploratory in nature, the results presented provide a snapshot of Portuguese community pharmacy and pharmacists. The different activities of DSRM yielded important results that may have implications for community pharmacy and pharmacists’ future that will be discussed in this section.

### Research findings

Regarding the results of each activity, some comments can be made. Firstly, the scenario exercise allowed to identify the main drivers to the diffusion of new services: community pharmacists’ ability to innovate and develop new services is dependent not only on the “inner will” of individual pharmacy owners toward innovation, but also on the leadership of professional organizations such as the Pharmaceutical Society (OF) and the National Association of Pharmacies (ANF). Also, the economic context and pharmacy setting has to become more competitive, possibly through a liberalization of the pharmacy market or other legislative changes that would encourage innovators. More details on the methodology and comprehensive discussion of this scenario exercise can be found elsewhere [23].

The survey’s low response rate hinders our ability to make inferences for all Portuguese pharmacies. This low rate may also confirm the low interest in participating in pharmacy practices studies found in other recent studies [48, 49]. However, the results from the survey suggest that the potential to develop online services exists. All pharmacies are equipped with an information system and other technological infrastructures that can be further explored. However, pharmacy staff must be proficient in the use IST to support the provision of online pharmaceutical services, besides the evident need to acquire the technical skills that will allow and efficient and safe provision of pharmaceutical care. The conditions that have to be met in order to make online services ubiquitous in the country seem dependent of financial incentives and support from professional organizations, a finding that confirms the results from the scenario analysis.

The dispensing of medicines was found to be the most significant pharmaceutical service provided. No structured medication management service or any other disease management service was observed, in spite of the pharmacies claiming it was provided. This was an expected finding, and the main driver of the current research project. After the development of pharmaceutical care programs in the turn of the century, these programs have started to wither away, with many pharmacies dropping the provision of these programs [49]. The causes for this have long been discussed within the profession and the reasons range from the lack of payment for these services to the professional inertia regarding the adoption of new forms of practice [50, 51]. Also, another barrier commonly cited has been the “lack of time” reported by pharmacists [52, 53]. Analyzing our results, there seems to exist a perception gap about pharmacists’ free time. In reality, enough free time exists to provide at least one consultation per day. More importantly, the potential to find more time is there, if the management of community pharmacies is reorganized, with more clear roles for each of the professionals.

In the demonstration activity, pharmacists P also refer “lack of time” as the reason to under-recruit and not provide the necessary follow-up for the recruited users. Pharmacists overestimated the time needed to recruit patients referring that it would take at least 30 minutes per patient. In reality, after measures done by the researchers, the estimated time for recruitment was 18.32 minutes (about 60% of the pharmacists’ estimation) tested in 12 interventions at real time at pharmacy 1, 2 and 3. Moreover, one other reason provided for the low recruitment numbers was that pharmacists anticipated non-compliance with this type of intervention by their usual chronic patients, confirming the experience of other research projects in Portugal and elsewhere [16, 54]. To overcome this issues, innovative recruitment strategies must be considered and implemented in future research projects.

The results with Pharmacist “C”, providing the service in a community setting without a pharmacy infrastructure as support was a novelty. It has been advanced as a possible form of practice for future pharmacists [55]. What this experiment showed is that being available to capture patients’ interest and holding regular meetings are important activities to keep the patient engaged with his/her own disease management. The use of the personal profile and vital data graphics was an essential key to empower patients on their health self-management, enhancing their health condition perception allowing for better user-acceptance, medication adherence and healthy behaviors. Also of importance, the close monitoring and sharing of information allowed the detection of early signs of adverse reactions or potentially dangerous interactions between medicines and non-prescription products. By the end of the demonstration activity, the patients were so engaged that they asked if it was possible to continue to use the platform for data registration because they were already familiar with its functioning. These results show the high importance of continuing the research with new strategies to overcome barriers at community pharmacies.

### Implications for research

In this paper, the authors test DSRM as a method to develop new online services, in an iterative process that values end-users inputs. The use of design science to develop technological artifacts to use in healthcare has already demonstrated utility [56]. However, to our knowledge, the use of Peffers and colleagues [31] activities to develop a new health service is a novelty. These guidelines have been subject to discussion by experts in the field, [57] and hopefully this research work provides a new example of DSRM application for health service research. The DSRM activities were appropriate to study the contributions of the proposed ePharmacare online service to improve the interactions between pharmacists and patients. Moreover, the results from this DSRM cycle will inform the following research cycle.

Regarding the choice of methodology, the evaluation of the artifact must fulfil the four Österle principles for design-oriented information system research: abstraction, originality, justification and benefits [58]. The ePharmacare platform completely fulfils these four Österle principles by:

- Abstraction: ePharmacare may be used by pharmacists or by any other health professional ready to tackle chronic disease management and medicine utilization at both public and private health units. Data requirements are commonly available in any pharmacy or healthcare center, namely patient data, medicines interactions, etc.;
- Originality: ePharmacare was designed by a multidisciplinary team including healthcare professionals (e.g. pharmacists and GP) and patients, contributing to the creation of a system oriented to their real needs. For the first time the Dáder methodology for patient follow-up and pharmaceutical care was implemented in an online interactive system;
- Justification: According to the WHO, chronic diseases are a major worldwide cause of death and healthcare services burden [1]. ePharmacare is an online service to help prevent, manage and control chronic medication, validated by pharmacists and physicians;
- Benefits: the online service benefits healthcare organizations and the society in general by providing an integrated view of the patient and facilitating the work of healthcare professionals, namely pharmacists and general practitioners. It further contributes to more informed chronic diseases management, once it allows both pharmacists and physicians to check patient data in their specific contexts, speeding up the prescription decision process.

### Service modelling

**Fig. 4:**
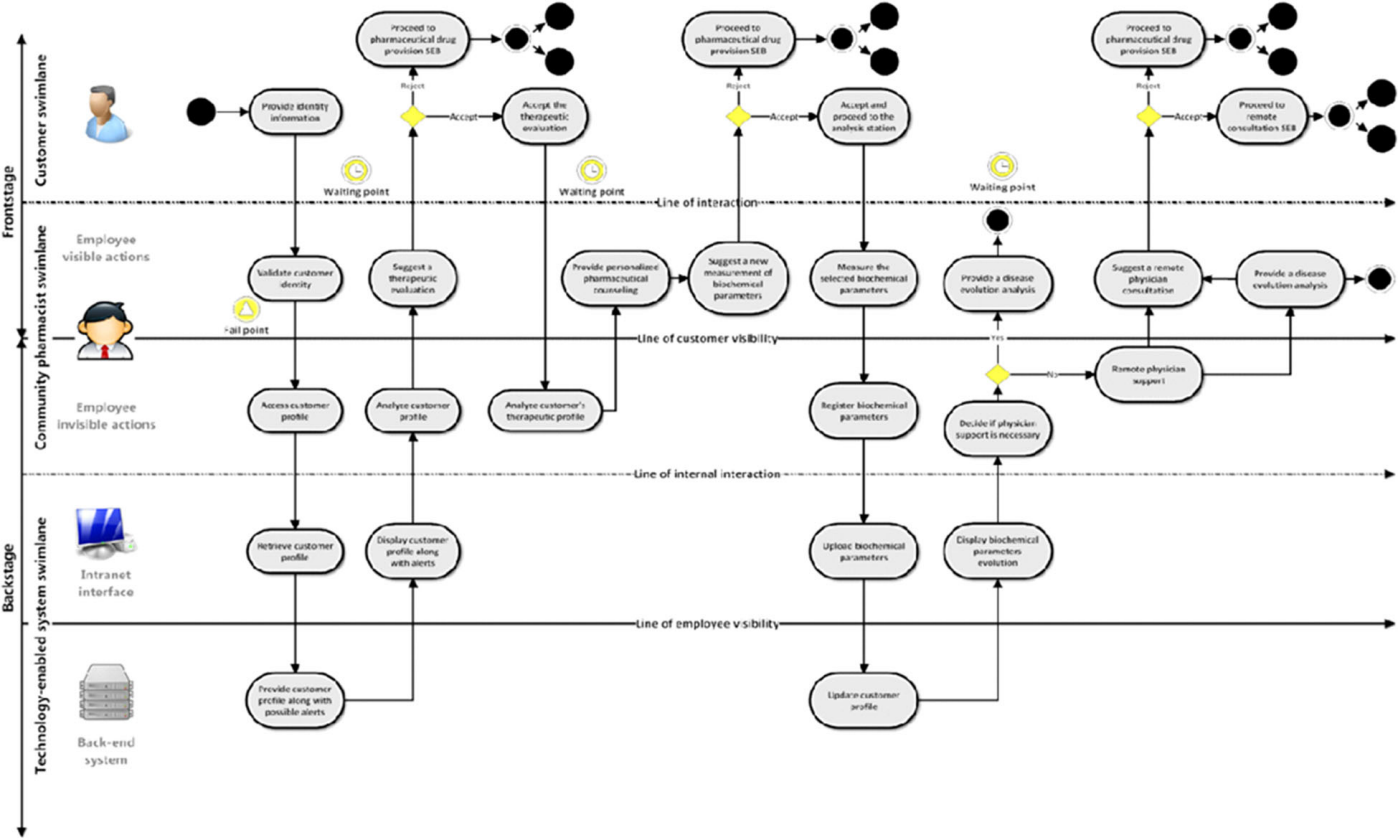
SEB for the new Disease Management service in community pharmacy (reproduced with author’s permission)[41]

The modelling stage at the end of activity 2 yielded an initial Service Experience Blueprint for the online service. The prosecution of all DSRM’s activities allowed the development of a new blueprint focusing on the architecture of a community pharmacy based disease management service. The SEB presented in Fig. 4 highlights the guidelines responsible for the service theater, identifying several actors (on the left) and the flow of possible actions throughout the service. This SEB also defines where the service takes place and how is the customer interacting with the service provider, as well as possible waiting points and fail points.

### Implications for pharmacy practice

Whether these professionals have the necessary skills to provide online pharmaceutical care services is an aspect that needs to be further explored. However, training provided on a web-platform with game-based learning, practicing with cases from daily activities, is a future possibility [59, 60]. Moreover, the social abilities that these professionals do have may provide the basis to improve the interaction experience with patients in a new online service [51, 61]. This can be important considering that customer relationships is one of the key aspects of the new services provided through the Internet.

One can argue that, for these developments to be beneficial, they should be considered in an integrated and longitudinal perspective of services provision, requiring service’s events registering, comprehensive analysis of the data and interactive dialogue with patients [62]. This perspective implies a new way of working in community pharmacies. Some authors refer that it is necessary to create a new organization at community pharmacies, not only to embrace pharmaceutical care, but also to incorporate it into their business models [63]. Surely community pharmacies and their professionals could play a more active role in chronic disease management within the healthcare system. Embracing the potential of the Internet to support the development of an extended role for community pharmacists is strategic [64].

**Fig. 5:**
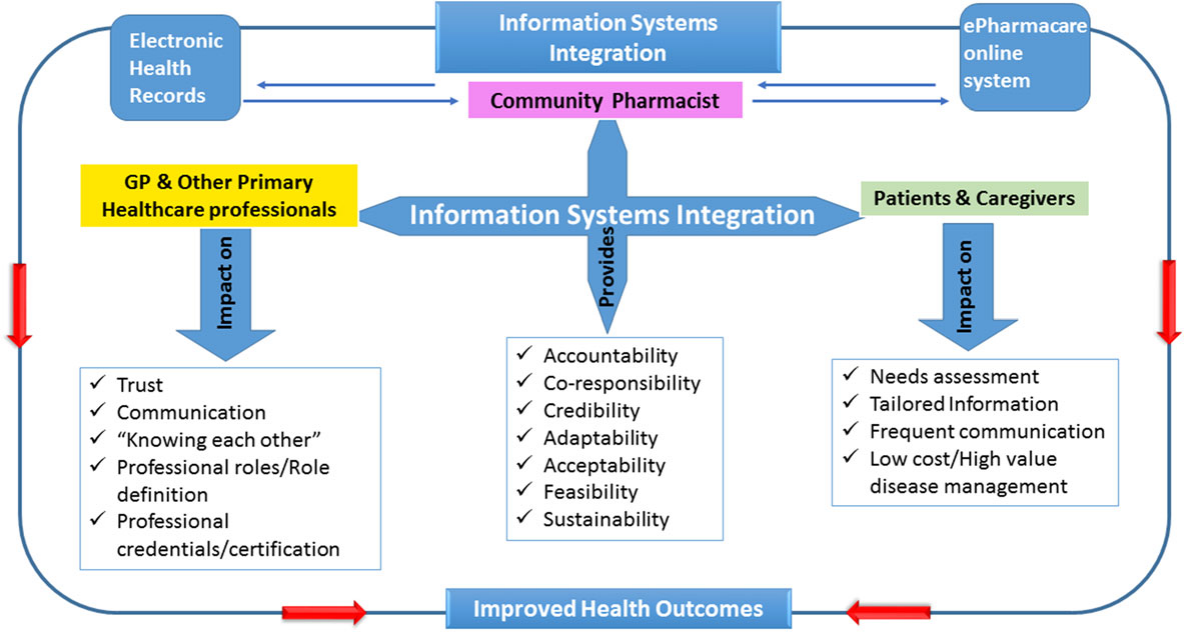
Conceptual model for the integration of Health Information Systems in Primary Healthcare

With the integration of primary healthcare and pharmaceutical services, data sharing portals such as the online platform developed for this study will receive data validated by primary healthcare professionals that are also useful for pharmacists’ provision of disease management services in the community. In a reciprocal way, the data gathered by patients and caregivers in the same portal, could be validated by community pharmacists before inclusion in the electronic health record used by primary care professionals. Figure 5 depicts a possible model for Health Information Systems integration. An integrated health information system that provides the stakeholders of disease management with mechanisms that assure accountability, credibility, acceptability among other dimensions, would have the potential to impact on several factors. On the professionals’ side, the dimensions of trust, communication, “knowing each other”, role definition and professional recognition and certification will surely benefit of such a system. On patients and caregivers’ side, an integrated system may improve professionals’ needs assessment, while providing tailored information through more frequent communication on a low cost service, yielding a high value disease management.

Innovation is a process implying the transformation of ideas about a perceived problem into a new product, process or service. Innovation in healthcare has usually a direct impact on the quality of care services and on the life of patients. The challenges posed by the rising prevalence of chronic diseases demand new strategies to innovation in health [65]. The ultimate goal of technology and innovation is to improve quality of life. Yet, all too often, introduction of new tools is decided by policymakers and experts without genuinely involving future users and those experiencing the potential risks [21]. However, good business model designs are likely to be context dependent, and both the design and implementation processes are likely to involve iterative processes [66].

The use of DSRM to implement an online pharmaceutical service offers a higher involvement to the stakeholders. This involvement showed the potential to develop a tailored information system. Furthermore, in the problem definition stage of the DSRM cycle presented, several issues relating to community pharmacists and pharmacies activities were identified. However, one must limit these findings to participant pharmacies in spite of the perception that these pharmacies provide a picture of the large majority of the country’s pharmacies. Caution is needed when interpreting the findings here reported.

The Dáder methodology to provide patient follow-up was chosen since it was the method used in the pioneer pharmaceutical care programs implemented in Portugal. All participant pharmacists who could provide pharmaceutical care were trained in this methodology. However, one can argue that other methodologies for patient follow-up namely, Medication therapy management (MTM), were available and could offer a valid alternative. The low number of participants in the demonstration stage also hinders the results, namely the estimation of the impact on health outcomes. In the next DSRM cycle, a better recruiting strategy must be defined to increase the number of participants, thus increasing the likelihood of having the sufficient number of participants to accurately assess the impact of online pharmaceutical services on the health outcomes of chronic patients. As it is an objective of a good DSRM study, a new cycle is being prepared to further improve the online service, this time focusing on improving the communication between pharmacists and physicians.

## Conclusion

The recent evolution of IST (e.g. website and chronic care sensors) is an important opportunity towards the integration of new roles for community pharmacists (within the healthcare system), while allowing a more active role from patients in their disease management. The ePharmacare platform presents a possible turning point in the way business is done within community pharmacies, by reaching out to new channels and shifting the focus from the sale of products to the provision of services.

The use of DSRM helped implementing an online pharmaceutical service through a higher involvement of the stakeholders in a comprehensive way. Moreover, in the problem definition stage of the DSRM cycle presented, several issues relating to community pharmacists and pharmacies activities were identified. The patterns of pharmacist time usage, the costing studies and the patterns of service provision and demand, reflect a need to reorganize pharmacies management and acquire additional skills in order to enable online pharmaceutical services provision.

The ePharmacare platform is able to offer pharmacies a way to better monitor their patients and therefore increase the quality of their therapy. By bringing to life a needed tool, the platform fulfills the detected need for IST in pharmaceutical services provision, offering patients a new way to interact with their data and be part of their own therapy as active members on disease management.

The quality and usability of the platform is critical. But the platform is not everything. Developing and implementing online services takes properly trained and motivated professionals. Online pharmaceutical services need to be more integrated in the current daily practices and some communication and marketing efforts need to be done, to recruit and demonstrate value to the chronic patients. A rethinking of the community pharmacy business model in order to effectively and coherently integrate community pharmacies services into the future health system models, where patients will have an increasing role in disease management, is paramount.

## Abbreviations

AFP: Portuguese association of pharmacies
ANF: National association of pharmacies
CCM: Chronic care model
CP: Community pharmacist
DSRM: Design science research methodology
GP: General practitioner
IST: Information systems and technologies
NHS: National health service
OF: Pharmaceutical society
OTC: Over-the-counter medicines
SEB: Service experience blueprint
WHO: World health organization
